# Doctor-Shopping Behaviors among Traditional Chinese Medicine Users in Taiwan

**DOI:** 10.3390/ijerph120809237

**Published:** 2015-08-07

**Authors:** Ming-Hwai Lin, Hsiao-Ting Chang, Chun-Yi Tu, Tzeng-Ji Chen, Shinn-Jang Hwang

**Affiliations:** 1 Department of Family Medicine, Taipei Veterans General Hospital, No. 201, Sec. 2, Shi-Pai Road, Taipei 112, Taiwan; E-Mails: minghwai@gmail.com (M.-H.L.); htstar@gmail.com (H.-T.C.); sjhwang@vghtpe.gov.tw (S.-J.H.); 2 School of Medicine, National Yang-Ming University, No. 155, Sec. 2, Linong Street, Taipei 112, Taiwan; E-Mail: cytu1965@gmail.com; 3 Department of Family Medicine, Taipei Veterans General Hospital, Taoyuan branch, No. 100, Sec. 3, Cheng-Kung Road, Tao-Yuan 330, Taiwan

**Keywords:** traditional Chinese medicine, doctor shopping, complementary and alternative medicine, national health insurance, utilization

## Abstract

Doctor-shopping has caused an increase in medical expense, potential to receive duplicate medications, and suffer adverse drug reactions. We carried out a population-based retrospective study aimed at examining the user patterns of traditional Chinese medicine (TCM) ambulatory care in Taiwan. We retrieved complete TCM ambulatory visit datasets for the year 2007 from the National Health Insurance database in Taiwan. We defined the patients whose distribution of TCM physician numbers scored more than 97.5 percent (more than, or equal to, five TCM physicians) within one year as TCM doctor-shoppers. In total, 6,596,814 subjects (28.9%) paid TCM visits during that year. All 177,728 subjects (2.69%) who visited more than five (including) TCM physicians were classified as TCM shoppers. The most prevalent diagnostic grouping was upper respiratory infections (44.7%) and sprains and strains (44.0%). Men had a lower odds ratio (*OR*) among TCM shoppers than women (*OR* = 0.94, 95% confidence interval (*CI*) = 0.93–0.96). Younger people were less likely to be TCM shoppers than other people were. The *ORs* of TCM shoppers were higher among veterans and low-income patients (*OR* = 1.29 (1.23–1.35), and 1.33 (1.27–1.41)). In conclusion, health education on the potential of drug interactions and iatrogenic health risks incurred from doctor-shopping should be addressed to those high-risk patients.

## 1. Introduction

Doctor-shopping (or hospital-shopping), which means changing doctors (or hospitals) without professional referral for the same or similar illness conditions, is a worldwide phenomenon. The percentage of doctor-shopping varied and was dependent on the definition of doctor-shopping. There was no generally accepted definition of frequent attendance or doctor-shopping [1].

The literatures showed that doctor-shopping has caused increases in hospitalization cost [2], wastes in medical resources [3], and declines in continuous medical care [4]. Moreover, doctor-shopping patients are more likely to receive duplicate medications and suffer adverse drug reactions [5].

Due to the small proportion of the patient population receiving ambulatory care with a high number of physicians during the year, doctor-shopping would lead to greater medical expenses. Identifying and understanding this type of frequent users may be useful in developing strategies to adopt more effective intervention and reduce overuse [6].

There is a paucity of research in this area, with few articles published on this topic and available for the last decade, not to mention those especially on the topic of complementary and alternative medicine (CAM). Since the relevant research is rare in traditional Chinese medicine (TCM), the current study, therefore, tried to explore the associated influencing factors.

The use of CAM/TCM in Western countries has increased dramatically in the recent decades [7,8]. Owing to the different definitions of CAM, the types of CAM surveyed, and the survey methodologies and types of CAM reimbursed by insurance, it is difficult to compare the use frequency of CAM/TCM among countries.

The National Health Insurance (NHI) program in Taiwan started in 1995 and covers almost all inhabitants. Reimbursement for TCM has been included in the program since 1996. Only officially-accredited TCM physicians are qualified for reimbursement from the NHI, and are permitted to practice either in outpatient departments of hospitals or in clinics [9].

At the end of 2007, there were more than 2,700 TCM clinics contracted to the Bureau of NHI, providing TCM ambulatory care and services, such as oral medicine, acupuncture, and manipulative therapy. These qualified institutions obtain reimbursement from the NHI program in accordance with the official fee schedule [10,11].

Beneficiaries can choose between practitioners of Western medicine or comprehensive and easily accessible TCM services. In addition, they are allowed to visit primary-care clinics or hospitals without a referral. Approximately 26% of the twenty-three million people in Taiwan utilize National Health Insurance for TCM [11]. According to Taiwan’s NHI program, beneficiaries can choose multiple practitioners of TCM or/and Western medicine doctors freely. The widespread use of TCM among the population in Taiwan may not be surprising, since TCM has been developed in China for more than 2000 years and the ancestors of most Taiwanese were immigrants from China since the 17th century, onwards. These factors would all account for the high utilization of TCM [12]. Since all claim data are available to researchers in an electronic form, a large-scale survey can be feasibly conducted.

## 2. Methods

### 2.1. Database

The NHI program was initiated in Taiwan since 1995 and covers 22,803,048 beneficiaries at the end of 2007, equivalent to a coverage rate of 99.3%. In 1999, the Bureau of NHI began to release all claim data in electronic form to the public under the National Health Insurance Research Database (NHIRD) project (NHIRD; http://w3.nhri.org.tw/nhird/). The structure of the claim files is described in detail on the NHIRD website and other publication [10,13].

For privacy protection, the unique identifiers of the patients and institutions were scrambled cryptographically to assure anonymity, but the standard encrypting procedures made it possible to link those claims of the same patient within the NHIRD datasets.

### 2.2. Study Population

To analyze the utilization of TCM use in the population, we defined the patients whose distribution of TCM physician numbers scored more than 97.5 percent within one year as TCM doctor-shoppers [1]. We also investigated the other TCM users for comparative analysis. 

We obtained the complete TCM ambulatory visit datasets of 2007 (CM_CD2007.DAT: 34,792,480 records) from NHIRD, including the office-visit files and corresponding patients profiles for the year 2007 in Taiwan. The office-visit files recorded the dates of encounters, medical care facilities and specialties, patients’ genders, dates of birth, major diagnoses, and medical cost. We selected outpatient visits with physician’s diagnostic fee charged. Thus, patient’s visits for only acupuncture, trauma or orthopedic therapy without physician consultation were not included in this study. Although the concept of disease entities in TCM is quite different from that in Western medicine, TCM physicians are requested to code for office-visit claims with a diagnosis based on the International Classification of Disease, 9th Revision, Clinical Modification (ICD-9-CM) [13,14] with no more than three diagnostic codes at each visit when claiming reimbursement. Diagnoses at each TCM visit were reclassified using the single-level disease category of Clinical Classifications Software (CCS) for ICD-9-CM (https://www.hcup-us.ahrq.gov/toolssoftware/ccs/ccs.jsp).

The NHIRD includes a registry system for “Catastrophic Illnesses”. “Catastrophic illness” is a special identity in Taiwan’s insurance system to provide patients suffering from serious illness such as cancer, chronic autoimmune disease, and chronic organ failure a lower medical cost. Insured persons with major diseases can apply for a catastrophic illness registration card from the Bureau of NHI. We used variables of “catastrophic illness”, “farmer or fisherman”, and “veteran” from the NHIRD dataset to characterize socio-economical category. If the patients accept acupuncture, trauma and dislocation therapy, and have ever admitted in previous year, there are special annotations in the dataset. This study was exempted from full review by the Institutional Review Board because of the NHIRD’s de-identified nature (Institutional Review Board, Taipei Veterans General Hospital, No.: 2013-04-005E).

### 2.3. Statistical Analysis

We used Microsoft SQL Server 2012 (Microsoft Corp., Redmond, WA USA) for data linkage analysis and processing. Descriptive data, including the mean (standard deviation) and frequencies (percentage), were presented as continuous variables and discrete variables, respectively.

For data analysis, SPSS for Windows Version 15.0 (SPSS Inc., Chicago, USA) was employed. We used logistic regression to estimate the odds ratio (OR) as the measure of association of doctor shoppers. We conducted univariate analyses first; all variables that were significantly associated with TCM shoppers were included in multivariable logistic analysis. However, to avoid multicollinearity, the TCM visit counts were not included in the model.

Odds ratio estimates and 95% confidence intervals were calculated for different levels of significant variables as oppose to each variable’s corresponding reference level. A P value of less than 0.05 was considered statistically significant.

## 3. Results

Among the 22,803,048 valid beneficiaries of the NHI at the end of 2007 in Taiwan, 6,596,814 subjects (28.9%) paid TCM visits during that year. The average visit of TCM services, TCM clinics, and TCM physicians per patient in 2007 were 4.97 ± 6.63 (range 1–238), 1.38 ± 0.74 (range 1–74), and 1.66 ± 1.09 (range 1–82), respectively.

A total of 177,728 subjects (2.69%) who visited more than, or equal to, five TCM physicians in 2007 were classified as TCM shoppers (Table 1). Table 2 shows the comparison of patient profiles between TCM physician shoppers and non-shoppers. The average visit of TCM services and TCM clinics were both higher in shopper patients (16.5 ± 10.19 *vs.* 4.65 ± 6.18 visits, 3.61 ± 1.56 *vs.* 1.32 ± 0.60 clinics, *p* < 0.001).

**Table 1:** Distribution of patients in traditional Chinese medicine (TCM) ambulatory care according to the number of TCM physicians visited in 2007.

No. of Physicians Visited	No. of Patients	Percentage	Cum. Percentage
1	4,019,616	60.9%	60.9%
2	1,533,738	23.2%	84.2%
3	616,074	9.3%	93.5%
4	249,672	3.8%	97.3%
5	101,874	1.5%	98.9%
6	42,070	0.6%	99.5%
7	17,880	0.3%	99.8%
8	8009	0.1%	99.9%
9	3628	0.1%	99.9%
10	1844	0.0%	99.9%
11–20	2337	0.0%	99.9%
≥21	72	0.0%	100.0%
Total	6,596,814	100.0%	

**Table 2:** Patient characteristics of TCM shoppers and non-shoppers.

Characteristics	Non-Shopper (*n* = 6,419,086, 97.3%)	Shopper (*n* = 177,728, 2.7%)	*p*
Mean TCM visit /yr (±SD)	4.7 (±6.2)	16.5 (±10.9)	<0.001
Mean TCM clinic /yr (±SD)	1.3 (±0.6)	3.6 (±1.6)	<0.001
Mean TCM physician /yr (±SD)	1.6 (±0.8)	5.8 (±1.4)	<0.001
Sex			<0.001
Male	2,716,988 (42.3)	63,082 (35.5)	
Female	3,702,098 (57.7)	114,646 (64.5)	
Age (years)			<0.001
0–9	389,097 (6.1)	6520 (3.7)	
10–19	853,926 (13.3)	10,833 (6.1)	
20–29	1,183,604 (18.4)	32,718 (18.4)	
30–39	1,157,842 (18.0)	36,494 (20.5)	
40–49	1,130,294 (17.6)	37,661 (21.2)	
50–59	884,553 (13.8)	29,205 (16.4)	
60–69	439,766 (6.9)	13,606 (7.7)	
≥70	380,004 (5.9)	10,691 (6.0)	
Clinic Count			<0.001
1	4,778,940 (74.5)	9320 (5.2)	
2	1,289,201 (20.1)	30,439 (17.1)	
≥3	350,945 (5.5)	137,969 (77.6)	
Visit Count			<0.001
1–5	4,867,146 (75.8)	6120 (3.4)	
6–10	852,012 (13.3)	55,693 (31.3)	
11–30	626,623 (9.8)	98,073 (55.2)	
≥31	73,305 (1.1)	17,842 (10.0)	
Mean Outpatient department Cost (US$)			<0.001
<13.8	739,538 (11.5)	4004 (2.3)	
13.8–17.2	2,812,307 (43.8)	75,197 (42.3)	
≥17.2	2,867,241 (44.7)	98,527 (55.4)	
Special Socioeconomicstatus			
Low income	59,852 (0.9)	2638 (1.5)	<0.001
Farmer or fisherman	515,080 (8.0)	12,948 (7.3)	<0.001
Veteran	93,507 (1.5)	3923 (2.2)	<0.001
Catastrophic illness	224,244 (3.5)	8935 (5.0)	<0.001
Associated Medical Illness			
Admission in 2006	473,058 (7.4)	16,025 (9.0)	<0.001
Acupuncture	1,355,903 (21.1)	90,308 (50.8)	<0.001
Trauma	2,198,961 (34.3)	108,501 (61.1)	<0.001
Dislocation	5034 (0.1)	353 (0.2)	<0.001

*Note*: S.D.: standard deviation; US$1: New Taiwan Dollar (NTD) $30.0.

The mean age of shoppers and non-shoppers were 40.2 ± 17.7, and 36.9 ± 19.3. The proportion of younger patients (under 20s) among shoppers was lower than that among non-shoppers; however, the proportion of older patients (above 30s) among shoppers was higher than that among non-shoppers. In addition, shoppers were more likely to be female subjects than male subjects (female: shopper 64.5% *vs.* non-shopper 57.6%, male: shopper 35.5% *vs.* non-shopper 42.3%).

Three-quarters of non-shoppers had 1–5 TCM visits; however, 96.6% shoppers had more than five TCM visits in 2007. For all shoppers, 77.6% patients visited more than 3 clinics, whereas 75.8% non-shoppers just visited one clinic. The mean outpatient department (OPD) cost was US$19.8 ± 5.8 per TCM shopper visit and US$19.0 ± 7.0 per TCM non-shopper visit (calculated by an exchange rate of US$1 to NTD (New Taiwan Dollar) $30.0). 

Among the 32,813,217 TCM visits, 96.0% had one clinical diagnosis, 3.5% had two diagnoses, and 0.5% had three diagnoses, according to the ICD-9-CM coding system. The top 20 diagnostic groups among TCM ambulatory visits were listed in Table 3. The most prevalent diagnostic grouping among shoppers were other upper respiratory infections (44.7%); sprains and strains (44.0%); superficial injury, contusion (33.1%); spondylosis, intervertebral disc disorders, other back problems (30.4%); other connective tissue disease (29.5%), and other upper respiratory disease (27.4%); residual codes, unclassified (26.6%).

**Table 3:** Top 20 diagnostic groups among traditional Chinese medicine ambulatory visits in 2007.

Rank	Diagnostic Grouping (according to CCS)	No. (%)	Patients No. (%)	
		All visits (*n* = 32,813,217)	Shoppers (*n* = 177,728)	Non-Shoppers (*n* = 6,419,086)
1	Other upper respiratory infections	3,303,190 (10.1)	79,523 (44.7)	1,232,907 (19.2)
2	Other upper respiratory disease	2,421,278 (7.4)	48,666 (27.4)	746,633 (11.6)
3	Sprains and strains	2,165,912 (6.6)	78,204 (44.0)	1,273,543 (19.8)
4	Superficial injury; contusion	1,964,312 (6.0)	58,764 (33.1)	1,046,177 (16.3)
5	Other lower respiratory disease	1,840,173 (5.6)	47,510 (26.7)	712,954 (11.1)
6	Other gastrointestinal disorders	1,658,550 (5.1)	41,819 (23.5)	510,117 (7.9)
7	Spondylosis; intervertebral disc disorders; other back problems	1,638,392 (5.0)	53,962 (30.4)	733,601 (11.4)
8	Menstrual disorders	1,608,404 (4.9)	29,045 (16.3)	453,082 (7.1)
9	Residual codes, unclassified	1,554,857 (4.7)	47,254 (26.6)	496,701 (7.7)
10	Other connective tissue disease	1,390,896 (4.2)	52,458 (29.5)	705,963 (11.0)
11	Other disorders of stomach and duodenum	1,266,921 (3.9)	37,025 (20.8)	417,098 (6.5)
12	Headache; including migraine	1,162,674 (3.5)	40,080 (22.6)	445,121 (6.9)
13	Other non-traumatic joint disorders	1,036,148 (3.2)	40,359 (22.7)	509,645 (7.9)
14	Gastritis and duodenitis	575,717 (1.8)	16,187 (9.1)	209,792 (3.3)
15	Allergic reactions	446,920 (1.4)	9316 (5.2)	145,711 (2.3)
16	Cardiac dysrhythmias	420,260 (1.3)	13,681 (7.7)	132,341 (2.1)
17	Chronic obstructive pulmonary disease and bronchiectasis	424,853 (1.3)	9140 (5.1)	165,621 (2.6)
18	Other inflammatory condition of skin	399,705 (1.2)	9344 (5.3)	141,837 (2.2)
19	Conditions associated with dizziness or vertigo	370,819 (1.1)	13,848 (7.8)	138,711 (2.2)
20	Other skin disorders	36,7741 (1.1)	6743 (3.8)	112,284 (1.7)

Categorized using single level system disease category of Clinical Classifications Software for International, Classification of Diseases, Ninth Revision, Clinical Modification.

Table 4 shows the factors associated with TCM shoppers in the univariate and multivariate logistic regression. People with higher TCM clinic counts, and higher mean OPD cost, were more likely to be TCM shoppers. Men had a lower odds ratio (*OR*) of TCM shoppers than women (*OR* = 0.94, 95%*CI* = 0.93–0.96). Younger people (below 20s) were less likely to be TCM shoppers than older people.

**Table 4:** Factors associated with the traditional Chinese medicine shoppers in the univariate and multivariable logistic regression.

Characteristics	Univariate Analysis		Multivariable Analysis *	
	*OR* (95%*CI*) *p*-Value		*OR* (95%*CI*) *p*-Value	
Sex				
Male	0.75 (0.74–0.76)	<0.001	0.94 (0.93–0.96)	<0.001
Female	1.00 (reference)		1.00 (reference)	
Age (years)				
0–19	0.51 (0.50–0.52)	<0.001	0.84 (0.81–0.88)	<0.001
20–29	1.00 (reference)		1.00 (reference)	
30–39	1.14 (1.12–1.16)	<0.001	1.06 (1.02–1.09)	0.001
40–49	1.21 (1.19–1.22)	<0.001	1.22 (1.19–1.27)	0.005
50–59	1.19 (1.18–1.21)	<0.001	1.20 (1.18–1.22)	<0.001
≥60	1.07 (1.05–1.09)	<0.001	1.06 (1.02–1.09)	<0.001
Visit Count				
1–30	1.00 (reference)		——	
31–60	9.27 (9.11–9.44)	<0.001	——	
≥60	36.97 (33.84–40.39)	<0.001	——	
1	1.00 (reference)		1.00 (reference)	
2	12.10 (11.82–12.39)	<0.001	5.01 (4.89–5.13)	<0.001
≥3	201.51 (197.27–205.83)	<0.001	48.55 (47.48–49.64)	<0.001
Mean OPD Cost (US$)				
<13.8	1.00 (reference)		1.00 (reference)	
13.8–17.2	4.94 (4.78–5.10)	<0.001	1.71 (1.65–1.77)	<0.001
≥17.2	6.34 (6.15–6.55)	<0.001	2.13 (2.06–2.21)	<0.001
Special socioeconomic status				
Low income (ref = no) Yes	1.60 (1.54–1.67)	<0.001	1.33 (1.27–1.41)	<0.001
Veteran (ref = no) Yes	1.53 (1.48–1.58)	<0.001	1.29 (1.23–1.35)	<0.001
Farmer or fisherman Yes (ref = no)	0.90 (0.89–0.92)	<0.001	0.95 (0.93–0.97)	<0.001
Catastrophic illness Yes (ref = no)	1.46 (1.43–1.49)	<0.001	1.23 (1.19–1.27)	<0.001
Associated Medical Illness				
Admission in 2006 Yes (ref = no)	1.25 (1.23–1.27)	<0.001	1.12 (1.10–1.15)	<0.001
Acupuncture (ref = no) Yes	3.86 (3.82–3.89)	<0.001	1.58 (1.56–1.60)	<0.001
Trauma (ref = no) Yes	3.01 (2.98–3.04)	<0.001	1.73 (1.70–1.75)	<0.001
Dislocation (ref = no) Yes	2.55 (2.29–2.84)	<0.001	1.25 (1.08–1.44)	0.002

* The variables that were significantly associated with TCM shoppers were included in multivariable logistic analysis except we excluded the TCM visit count to avoid multicollinearity. OR: odds ratio; CI: confidence interval; US$1: NTD$30.0.

We also found that the ORs of TCM shoppers were lower among farmer or fisherman patients and were higher among veteran and low income patients (*OR* = 0.95 (0.93–0.97), 1.29 (1.23–1.35), and 1.33 (1.27–1.41), respectively). In addition, patients with catastrophic illness and patients ever admitted in previous year(s) were more likely to be TCM shoppers [*OR* = 1.23 (1.19–1.27), and *OR* = 1.12 (1.10–1.15)].

## 4. Discussion

This is the first study to provide a precise description for doctor-shopping among TCM patients in an entire population. Only with the aid of a computerized insurance reimbursement database could such a large-scale TCM utilization study be feasibly analyzed.

Several studies investigated the problem of doctor shopping, defined as patients who visit multiple physicians within a defined period [1]. In the present study, we applied the definition of doctor shopping with a distribution of physician numbers more than 97.5 percent, while seeking medical advice from more than, or equal to, five TCM doctors, such behavior is defined as TCM doctor-shopping in Taiwan. While comparing with the Western medicine (WM) users in Taiwan, 48.6% of WM patients seek medical advice from more than five WM doctors, and 5.6% of WM patients seek medical advice from more than 12 TCM doctors during one year. Thus, seeking medical advice from more than 13 WM physicians per year is defined as WM doctor-shopper [10].

Chi *et al.* suggested that patients with a regular source of care for Chinese medicine, as well as a preference for Chinese medicine, are two predictors for TCM users [9]. Zwaan *et al.* proposed that most patients perceived Western medicine as more effective and held higher expectations from the Western drugs [14]. These may be the reasons why patients change Western medicine doctors more frequently than TCM doctors do. However, the possibility of overuse deserves further attention [15].

In this study, the multivariable analysis showed significant difference between the two groups of patients with regard to sex, age, mean medical cost, and other demographic profiles. Wang’s study on doctor-shopping behavior with upper respiratory tract infection in Taiwan showed a reverse U-shaped relationship with the patient age (the highest in age 18-34 years) [16]; our result showed that subjects between 40 and 49 years of age comprised the largest *OR* of doctor-shopping than other age groups (*OR* = 1.22, 95%*CI* = 1.19–1.27). Our findings are also consistent with the previous reports from Demers [2] that the shopper patients received 10 times more medical services than the overall patient population, and the mean cost per patient for ambulatory care was also higher.

The use of CAM/TCM in Western countries is usually not covered by insurance. Since TCM is reimbursed by NHI in Taiwan, our study would appear to be less affected by the socio-economic status of the subjects [8]. Other characteristics of doctor-shoppers were veterans, low income, and serious health status. With regard to the above demographic factors, the proportion of patients among shoppers was higher than that among non-shoppers. These effects were robust after multivariate adjustment and testing for effect modification. It may imply that doctor shoppers are mostly from the disadvantaged minority groups. We hereby suggest an in-depth exploration on the impact of doctor-shopping behavior in order to improve medical qualities. 

Hagihara *et al.* found that doctor-shopping patients had a high lifetime prevalence of mental disorders by using questionnaire and interview surveys [6]. The lifetime prevalence of DSM-III somatization disorders was significantly higher in the two study groups. Kasteler suggested that factors related with the tendency to shop for doctors in specific groups were a lack of confidence in doctors’ competence, unwillingness of doctors to spend time talking with patients, hostile feelings toward doctors, high cost of services, inconvenience of location and hours, and unfavorable attitudes toward doctors’ personal qualities [17,18].

These results reveal that the risk factors for doctor-shopping are strongly associated with chronic conditions and fragile doctor-patient relationships. Hagihara *et al.* found that the patient's inability to understand a doctor’s explanation on the treatment itself, which results from a large gap between the perceptions of the patient and those of the doctor [6]. They thought it to be the most significant predictor of doctor-shopping behavior, and suggested that in the case of insufficient explanations provided, doctors may be able to prevent doctor-shopping behavior by offering relatively thorough explanations on the treatment. Therefore, we emphasize on the importance of accurate explanations and maintenance of good doctor-patient relationships by physicians providing care. We also suggest that doctors should notice the patient’s medical history as for how many prior visits have been made with the same complaint.

Studies have shown that prescription drug monitoring programs (PDMPs) decrease the diversion and doctor-shopping [19]. Through electronic databases, health care providers can stop patients from doctor-shopping. In Taiwan, the Bureau of National Health Insurance (BNHI), which was renamed as National Health Insurance Administration (NHIA) in 2013, adopted smart cards (or NHI-IC cards) as health cards, and such a card carries the information of the patient's prescribed medications from all hospitals nationwide. Hsu conducted a pilot study for online detection of potential duplicate medications *via* a computerized physician order entry (CPOE) system to identify duplicate medications from multiple hospitals and, hence, enhance patient safety across hospital boundaries [5]. Online detection of potential duplicate medications for outpatient visits using national health insurance smart cards may help physicians detect potential duplicate medications for frequent doctor-shopping patients.

The study had some limitations. With datasets from the NHIRD, only officially-recognized TCM treatments that were reimbursed under the NHI program in Taiwan could be studied; thus, the results did not completely reflect the real use of TCM. People in Taiwan can buy herbal remedies from TCM pharmacies without seeing TCM physicians; therefore, out-of-pocket use of TCM herbs was not recorded. If we add up the self-paid TCM treatments, which are not included in the reimbursement data, it is quite possible for the frequency to rise. However, due to database limitations, this study only used one year’s data for analysis. There are possible selection biases in the present study, such as overt selection bias and hidden selection bias. Some patients may suffer from a disease and seek intensive ambulatory care in the study year only. Some may not be classified as doctor-shoppers just because they are not frequent users in the study year. We did not analyze whether the subjects are doctor-shoppers for both TCM and non-TCM doctors and whether the numbers of visits are the same between these two “types” of medical treatment.

The major strength of this study is its population-based and record-based nature, which assured that the results are rarely affected by selection or recall biases. In addition, the current analysis has a large sample size, which provides the study with high statistical power and precision.

## 5. Conclusions

TCM is one of the mainstream medicines among people in Taiwan and there are increasingly more people in other countries who accept it. This study provides relevant information for further studies. In conclusion, patients should be warned about the potential of (herbal) drug interactions and the iatrogenic health risks incurred from doctor-shopping. Health education programs are needed to modify unrealistic views on quality care. In the future, studies are suggested to investigate the underlying factors of doctor-shopping and the impact on the depletion of healthcare resources for health policy makers to build a better health delivery system.
